# A systematic review and narrative synthesis of interventions for uncomplicated obesity: weight loss, well-being and impact on eating disorders

**DOI:** 10.1186/s40337-017-0143-5

**Published:** 2017-05-01

**Authors:** Tina Peckmezian, Phillipa Hay

**Affiliations:** 1 National Eating Disorders Collaboration, Sydney, Australia; 2 Foundation Chair of Mental Health and Centre for Health Research, School of Medicine, Western Sydney University, Parramatta, Australia

**Keywords:** Obesity, Eating disorders, Treatment, Review

## Abstract

**Objective:**

Most weight loss research focuses on weight as the primary outcome, often to the exclusion of other physiological or psychological measures. This study aims to provide a holistic evaluation of the effects from weight loss interventions for individuals with obesity by examining the physiological, psychological and eating disorders outcomes from these interventions.

**Methods:**

Databases Medline, PsycInfo and Cochrane Library (2011–2016) were searched for randomised controlled trials and systematic reviews of obesity treatments (dietary, exercise, behavioural, psychological, pharmacological or surgical). Data extracted included study features, risk of bias, study outcomes, and an assessment of treatment impacts on physical, psychological or eating disorder outcomes.

**Results:**

From 3628 novel records, 134 studies met all inclusion criteria and were evaluated in this review. Lifestyle interventions had the strongest evidence base as a first-line approach, with escalation to pharmacotherapy and bariatric surgery in more severe or complicated cases. Quality of life was the most common psychological outcome measure, and improved in all cases where it was assessed, across all intervention types. Behavioural, psychological and lifestyle interventions for weight loss led to improvements in cognitive restraint, control over eating and binge eating, while bariatric surgery led to improvements in eating behaviour and body image that were not sustained over the long-term.

**Discussion:**

Numerous treatment strategies have been trialled to assist people to lose weight and many of these are effective over the short-term. Quality of life, and to a lesser degree depression, anxiety and psychosocial function, often improve alongside weight loss. Weight loss is also associated with improvements in eating disorder psychopathology and related measures, although overall, eating disorder outcomes are rarely assessed. Further research and between-sector collaboration is required to address the significant overlap in risk factors, diagnoses and treatment outcomes between obesity and eating disorders.

**Electronic supplementary material:**

The online version of this article (doi:10.1186/s40337-017-0143-5) contains supplementary material, which is available to authorized users.

## Plain English Summary

Obesity and eating disorders are often viewed as distinct problems on opposite ends of the weight spectrum, but in actuality, they share a number of risk and protective factors and frequently co-occur. Individuals with binge eating disorder are often overweight, and individuals that are overweight are at an increased risk of developing eating disorders. Despite this, weight loss interventions usually focus on physical factors, such as changes in weight or body composition, to the exclusion of other factors, such as psychological well-being or eating disorders.

In this review, we conduct a detailed examination of published literature on the range of available interventions for overweight and obesity, taking physical, psychological and eating disorders outcomes into equal consideration. ‘Lifestyle’ interventions that include exercise, dietary and behavioural components have the strongest evidence as a first-line approach, while obesity (bariatric) surgery is the most effective intervention in terms of the amount of weight lost. Quality of life is not commonly measured, but where reported it tends to improve alongside weight loss. Interventions that included behavioural or psychological components had the most positive impacts on eating disorders psychopathology and related measures, although these outcomes were only reported in a small number of studies.

## Background

Obesity and eating disorders are significant public health concerns that are associated with a range of adverse physical and psychological outcomes. In Australia, more than 60% of adults and 25% of children and adolescents are overweight or obese [1, 2], with an additional 16% presenting with disordered eating behaviours or eating disorders [3]. The rate of both eating disorders [4] and obesity [2] is increasing in the Australian population, and recent evidence indicates that the rate of comorbid obesity and eating disorder behaviours has increased more rapidly than either disorder alone [5]. Individuals with comorbid obesity and eating disorders face the added difficulty of receiving care for both the medical complications associated with obesity and the psychosocial impairments associated with eating disorders [5].

The reasons for such a large increase in comorbid obesity and eating disorders in a relatively short period of time are unclear, but media and weight-reduction campaigns have been suggested as precipitating factors [5]. Media exposure may increase sociocultural pressures to attain an ideal body image and contribute to body dissatisfaction [6–8], and community-based weight-reduction campaigns may inadvertently stigmatise the individuals they intend to help [9, 10] by encouraging dieting and physical exercise as a means to attain an ideal body weight or shape [11–13].

A growing body of evidence highlights the significant shared space between disorders at both ends of the weight spectrum. Obesity and eating disorders share a number of risk factors that apply to a broad range of eating- and weight-related problems [14–16]. These include (i) individual factors such as dieting, unhealthy weight-control behaviours, weight and shape concerns, and self-esteem issues; (ii) social factors, such as parental and peer weight and shape related behaviours, including bullying and histories of abuse; and (iii) societal factors, such as sociocultural norms, media exposure and weight discrimination. Collectively, these factors place enormous pressure on individuals to conform to an ideal weight and shape, and contribute to body dissatisfaction that is a predictor of both eating disorders and excessive weight gain [17].

Overweight individuals are at increased risk of disordered eating and eating disorders compared with the general population [18]. At the same time, individuals with binge eating disorder (BED) and individuals who use unhealthy weight-control practices (e.g. fasting, purging and diet pills), such as those with bulimia nervosa (BN), are at increased risk of overweight and obesity [19–22]. Further, individuals with comorbid BED and obesity are at increased risk of weight gain and related complications [23], and experience a higher rate of medical problems [24] and depression [25] than obese individuals without BED. Individuals with eating disorders are more than twice as likely to contact health professionals or weight loss centres for weight reduction assistance [4] than they are to seek treatment specifically for their eating disorder [4]. This raises the concern that interventions targeting weight loss may exacerbate or contribute to the development of disordered eating or eating disorders [26] by encouraging behaviours that increase focus on body shape and weight [27].

Since the vast majority of weight loss intervention research focuses on weight as the primary outcome, often to the exclusion of other physiological or psychological measures, the potential impact of weight loss interventions on eating disorders and overall wellbeing is unclear. Therefore, this systematic review aims to provide a holistic evaluation of the effects from weight loss interventions for individuals with obesity, assessing physiological, psychological and eating disorders outcomes. Due to the large volume of literature on the topic, we constrained our search to cases of uncomplicated obesity – that is, individuals who were otherwise healthy, and with the exception of BED, had no physical or psychological comorbidities. A synthesis of outcomes is then used to identify gaps in the literature and guide our recommendations for future research and practice that encourages an integrated approach to the prevention and management of obesity and eating disorders.

## Methods

This review was conducted in accordance to the PRISMA guidelines for systematic reviews [28]. Searches of the electronic databases Medline, PsycInfo and Cochrane Library were conducted in February 2016 using both medical subject headings (MeSH) and equivalent free text searches for terms pertaining to obesity (overweight, overnutrition, hyperphagia), obesity interventions (exercise, diet, psychological, pharmacological, behavioural and surgical), and eating disorders (anorexia nervosa, bulimia nervosa, binge eating disorder, other specified feeding and eating disorders).

### Study selection

We considered peer-reviewed systematic reviews (SRs) and randomized controlled trials (RCTs) published in 2011 or more recent, or in the case of SRs, an original or updated search date of 2011 or more recent. These criteria were chosen because of the large volume of available literature, because older RCTs will be evaluated within recent systematic reviews, and because recent systematic reviews eclipse non-recent systematic reviews. We excluded non-English studies, studies reporting primarily qualitative or methodological data, studies focused on preventative interventions and studies in which the outcomes of an overweight or obesity intervention were not the main focus of the study.

The target sample included overweight or obese males and females ranging in age from 12–65 years. Adolescents (12–18 years) were assessed separately from adults (18–65 years). Because of prominent differences in treatment strategies, paediatric and elderly patients were excluded from consideration. The primary interventions evaluated in this review were dietary, exercise/physical activity, psychological/behavioural, pharmacological and surgical interventions, while the primary outcome measures were standardized anthropometric measures (e.g., body mass index (BMI) and body weight), standardized body composition measures (e.g., body fat mass), psychological, eating disorder and quality of life measures, and reports of adverse treatment effects.

### Assessment of studies

All included studies were assessed first individually and second collectively (by TP and SB, see acknowledgements) according to treatment intervention. Study assessment included appraisal of study quality, clinical impact and consistency. Each assessment item was scored as high, moderate or low by one of two reviewers, and a subset of studies (10%) was scored by both reviewers to assess accuracy. Disagreements were resolved by discussion.

The Overview Quality Assessment Questionnaire (OQAQ) [29] and the Jadad Scale [30] were used to assess study quality and risk of bias for SRs and RCTs, respectively [see Additional file 1]. The OQAQ includes items that address the suitability of the search methods, study selection, data assessment and pooling, while the Jadad scale includes items that address the suitability of randomisation, blinding, and procedures to account for withdrawals and dropouts. The clinical impact rating was derived by assessing the statistical precision, effect size, and relevance to patients compared with other treatments or no treatment, based on the outcomes reported by the study author (i.e., rather than based on meta-analyses). Finally, consistency was assessed for SRs based on the consistency of included RCTs (adapted from NHMRC guidelines [31]) and the statistical heterogeneity of included studies (when this data were available).

Following the assessment of each individual SR and RCT, the evidence was evaluated for each intervention, taking into account the number, quality, clinical impact and consistency of included studies. Each factor was assigned a rating of poor, satisfactory, good or excellent based on the degree to which relevant criteria (adapted from the NHMRC Body of Evidence Matrix [31]) [see Additional file 1] were fulfilled. Table 1 presents a summary of all interventions evaluated in this review, along with a summative assessment of the breadth of the evidence base (quality/quantity of studies), consistency and clinical impact of included studies.

**Table 1:** Summary of interventions: Evidence base, consistency and clinical impact

Intervention	Intervention: Specific	Evidence Base	Consistency	Clinical Impact
Dietary	***Weight loss outcomes***	Satisfactory	Good	Satisfactory
	Increased fruit & vegetables	Satisfactory	N/A	Poor
	Low energy diets (overall)	Satisfactory	Good	Poor
	Low calorie	Satisfactory	Poor	Poor
	High protein/low carbohydrate	Poor	N/A	Poor
	Portion controlled meals	Satisfactory	Satisfactory	Satisfactory
	***Mental health outcomes***	Poor	N/A	Satisfactory
	***Eating disorders outcomes***	N/A	N/A	N/A
Exercise & Physical Activity	***Weight loss outcomes***	Good	Good	Good
	Aerobic + resistance	Satisfactory	Good	Good
	High intensity interval training	Satisfactory	Poor	Good
	Pilates	Poor	N/A	Poor
	***Mental health outcomes***	Poor	N/A	Satisfactory
	***Eating disorders outcomes***	N/A	N/A	N/A
Behavioural & Psychological	***Weight loss outcomes***	Good	Good	Satisfactory
	Behavioural weight loss	Satisfactory	Good	Good
	Cognitive behavioural therapy	Good	Poor	Satisfactory
	Mindfulness based therapies	Satisfactory	Poor	Poor
	Acceptance based therapy	Satisfactory	N/A	Satisfactory
	Motivational interviewing	Satisfactory	Poor	Satisfactory
	Self-monitoring programmes	Satisfactory	Satisfactory	Satisfactory
	***Mental health outcomes***	Satisfactory	Poor	Satisfactory
	***Eating disorders outcomes***	Satisfactory	Good	Good
Lifestyle Interventions	***Weight loss outcomes***	Satisfactory	Satisfactory	Good
	Diet + Exercise	Satisfactory	Good	Good
	Diet + Behavioural	Satisfactory	Poor	Poor
	Behavioural + Exercise	Satisfactory	Satisfactory	Good
	Behavioural + Exercise + Diet	Satisfactory	Good	Good
	***Mental health outcomes***	Satisfactory	Poor	Poor
	***Eating disorders outcomes***	Satisfactory	N/A	Satisfactory
Pharmacotherapy	***Weight loss outcomes***	Excellent	Good	Good
	*Pharmacotherapy only*	Excellent	Good	Good
	Anorectics	Good	Excellent	Good
	Antidiabetics	Satisfactory	N/A	Good
	Antidepressants	Good	Good	Good
	Anticonvulsants	Satisfactory	N/A	Satisfactory
	Vitamin D & calcium	Satisfactory	Poor	Poor
	Sodium alginate	Satisfactory	N/A	Poor
	Fatty acid supplements	Good	Good	Poor
	Dietary protein	Satisfactory	N/A	Poor
	Probiotics	Satisfactory	N/A	Satisfactory
	Glucomannan supplement	Satisfactory	Good	Poor
	Chromium supplement	Satisfactory	N/A	Good
	Yeast hydrolysate	Satisfactory	N/A	Satisfactory
	*Gynostemma pentaphyllum*	Satisfactory	N/A	Poor
	Green tea extract	Good	N/A	Poor
	*Pharmacotherapy + Other*	Good	Satisfactory	Satisfactory
	Pharmacotherapy + Diet	Satisfactory	Satisfactory	Satisfactory
	Pharmacotherapy + Exercise	Satisfactory	N/A	Poor
	Pharmacotherapy + Behavioural	Good	Good	Good
Pharmacotherapy	Pharmacotherapy + Diet + Exercise + Behavioural	Good	Poor	Poor
	***Mental health outcomes***			
	*Single interventions*	Satisfactory	Poor	Poor
	*Combined interventions*	Satisfactory	Poor	Poor
	***Eating disorders outcomes***			
	*Single interventions*	Satisfactory	Poor	Poor
	*Combined interventions*	Poor	N/A	N/A
Bariatric Surgery	***Weight loss outcomes***	Excellent	Good	Excellent
	Roux-en-Y gastric bypass	Good	Satisfactory	Good
	Laparoscopic sleeve gastrectomy	Good	Good	Good
	Laparoscopic gastric banding	Satisfactory	Satisfactory	Good
	Duodenal switch	Satisfactory	Satisfactory	Good
	***Mental health outcomes***	Excellent	Good	Good
	***Eating disorders outcomes***	Excellent	Satisfactory	Good
Other Interventions	***Weight loss outcomes***	Satisfactory	Poor	Poor
	Acupressure	Poor	Good	Satisfactory
	Acupuncture	Satisfactory	N/A	Good
	Bright light therapy	Satisfactory	N/A	Poor
	Hot bathing (+ Diet + Exercise)	Satisfactory	Poor	Satisfactory
	***Mental health outcomes***	Poor	N/A	Poor
	***Eating disorders outcomes***	N/A	N/A	N/A
Weight loss interventions for adolescents	***Weight loss outcomes***	Good	Satisfactory	Satisfactory
	Diet	Satisfactory	Good	Satisfactory
	Exercise (aerobic)	Satisfactory	Poor	Satisfactory
	Behavioural (cognitive behavioural therapy)	Satisfactory	N/A	Satisfactory
	Pharmacological (antidiabetics)	Satisfactory	Good	Good
	Surgical	Good	Satisfactory	Satisfactory
	Lifestyle	Satisfactory	Good	Satisfactory
	Lifestyle + Pharmacological	Satisfactory	N/A	Satisfactory
	***Mental health outcomes***	Satisfactory	Good	Satisfactory
	***Eating disorders outcomes***	Satisfactory	Good	Good

Note: The evidence base reflects the quantity and quality of available studies: Excellent = >1 SRs or >2 RCTs with low risk of bias; Good = 1–2 RCTs or 1 SR with low risk of bias; Satisfactory = SRs or RCTs with moderate risk of bias; Poor = SRs or RCTs with high risk of bias. Consistency reflects the degree to which study outcomes are consistent with each other: Excellent = all studies consistent; Good = mostly consistent; Satisfactory = some inconsistency; Poor = inconsistent; N/A = only 1 RCT. The clinical impact reflects the statistical and clinical significance of study findings: Excellent = very large; Good = substantial; Satisfactory = moderate; Poor = slight or restricted. The bolded terms (weight loss, mental health and eating disorders outcomes) reflect a summary evaluation of all interventions in each broader category

## Results

**Fig. 1:**
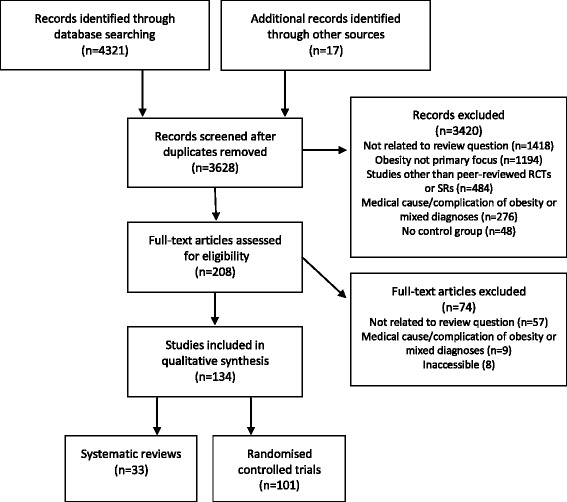
Flow chart overview of literature search process

The search strategy yielded a total of 3628 citations after removal of duplicates. Following exclusion of studies that did not meet inclusion criteria, 134 studies remained, of which 33 were SRs and 101 were RCTs (Fig. 1). Additional files summarize the study features, risk of bias, study outcomes, clinical impact and citation information of all SRs and RCTs included in this review [see Additional file 2 and 3 for SRs and RCTs, respectively]. The most common measure of intervention success was the amount of weight lost. For a summary of interventions included in this review, see Additional file 4.

### Lifestyle interventions

Lifestyle interventions were evaluated in 2 SRs [32, 33] and 11 RCTs [34–44] and were found to be largely effective at improving body weight and related measures in overweight and obese individuals. Specifically, interventions that included diet plus exercise or diet plus exercise plus behavioural/psychological components had consistently positive outcomes. Mental health was assessed in a single SR [33], which reported quality of life improvements following a lifestyle intervention. A single RCT [40] evaluated eating disorders outcomes from a lifestyle intervention for weight loss. In this study, cognitive behavioural therapy (CBT) was prescribed to obese patients with BED in combination with a low-energy-density diet or general nutrition counselling. Both groups had significant reductions in binge eating, and at 12-month follow-up, had significant improvements in behavioural and attitudinal features of BED. No adverse effects were reported.

### Dietary interventions

Two SRs [45, 46] and 5 RCTs [47–51] evaluated low-energy diets for weight loss. Weight loss was reported in studies in which participants received very-low-calorie ketogenic diets, high-protein diets or portion-controlled diets, although in most cases this effect was not sustained over longer (12-month) follow-up periods. Increased fruit and vegetable consumption, low carbohydrate diets and a ready-to-eat cereal snack replacement were ineffective as weight loss interventions. Physical adverse effects were reported in only a single study in which participants received very-low-calorie ketogenic diets, and these effects ceased when a normal diet was resumed. Only a single RCT [48] assessed mental health outcomes associated with dietary interventions for weight loss. This study compared the meal replacement Optifast to a conventional reduced-fat diet, and reported significant quality of life improvements in both groups. There were no studies reporting eating disorders outcomes from dietary interventions for weight loss.

### Exercise

Exercise was prescribed both on its own and in combination with behavioural and dietary interventions within a lifestyle intervention. The intensity, frequency and duration of the program were important parts of the exercise prescription. Weight loss was reported in one SR [52] and all but one of the 8 RCTs [53–60] evaluating exercise interventions. A number of these studies also reported favourable changes in body composition, such as reductions in body fat, and no adverse effects were reported. Only a single RCT [58] assessed mental health outcomes associated with exercise interventions for weight loss. In this study, quality of life scores improved in moderate- and high-intensity exercise groups compared with no-exercise controls. There were no studies reporting eating disorders outcomes from exercise and physical activity interventions for weight loss.

### Behavioural and psychological Interventions

Weight loss was reported, to varying degrees, in the majority of the SRs [61–67] and RCTs [68–81] evaluating behavioural and psychological interventions. Interventions producing positive effects on weight loss included lifestyle counselling, self-help CBT, and a number of technology-based behavioural interventions, while mixed outcomes occurred following behavioural weight loss or management programmes (including commercially available programmes), mindfulness based interventions and motivational interviewing interventions. A subset of studies targeted obese patients with binge eating disorder. Of these, motivational interviewing yielded reductions in weight, while CBT had mixed outcomes depending on the type: standard and virtual-reality enhanced CBT, but not self-help CBT, produced weight loss. No adverse effects were reported.

Mental health outcomes were evaluated in three RCTs. One RCT [70] reported quality of life improvements in participants in standard and acceptance-based behavioural interventions, with no significant difference between groups. Two RCTs assessed changes in depression symptoms with weight loss treatments targeting obese patients with binge eating disorder. One study [81] reported an improvement in depression symptoms with a motivational-interviewing weight loss intervention that was statistically greater than controls, while the second [72] reported a similar improvement with a self-help CBT intervention, however an improvement was observed in the control group as well.

Eating disorders outcomes were evaluated in 5 RCTs, and all reported positive outcomes. One study [77] reported improved cognitive restraint and control over eating after participation in a 3-year lifestyle intervention which included healthy diet and exercise counselling. Four studies reported reductions in binge eating episodes following standard CBT [71], self-help CBT [72], virtual-reality enhanced CBT [69], motivational interviewing [81] and behavioural weight loss [61]. Behavioural weight loss and standard CBT also led to significantly greater remission from binge eating compared with participants receiving a control intervention.

### Pharmacological interventions

Seven SRs [82–88] and 33 RCTs [89–122] evaluated exclusively pharmacological interventions for weight loss, and 13 RCTs evaluated pharmacotherapy as an adjunct to other interventions. Registered medicines, including anorectics, antidepressants, anticonvulsants and antidiabetics, were highly effective at reducing weight. However, all but one study also reported adverse effects that ranged from mild to severe. In contrast, listed “complimentary” medicines were less effective (less than half of all studies reported positive weight loss outcomes) and also reported fewer adverse effects (less than half of all studies reported adverse effects).

Pharmacotherapies delivered alone led to improvements in quality of life, depression and anxiety (diethylpropion, fenproporex, mazindol, fluoxetine and sibutramine) as well as mood, fatigue and confusion (lipid dietary supplement), although one substance (the anticonvulsant zonisamide) led to a worsening of anxiety, depression, attention, concentration and memory.

Pharmacotherapies delivered as an adjunct to behavioural and psychological interventions led to improvements in quality of life (bupropion/naltrexone plus behavioural modification [111]) and depression symptoms (self-help CBT plus sibutramine [123]).

Eating disorders were evaluated in two RCTs assessed. The first study [114] prescribed the antidepressant medication bupropion to obese women with BED and reported that medication did not improve binge eating, food cravings or associated eating disorders features relative to placebo. The second study [106] was the first to prescribe the endocannabinoid medication rimonabant to obese patients with BED. The treatment group had a significantly greater reduction in scores on the Binge Eating Scale compared with placebo controls; however, this change was not likely to be clinically significant.

### Bariatric surgery

Nine SRs [124–132] and 6 RCTs [133–138] evaluating exclusively surgical interventions for weight loss were included in this review. Most SRs (7 of 9) assessed outcomes from multiple surgical procedures, and a subset of these pooled outcomes across procedures. Compared with non-surgical interventions, bariatric surgery produced significantly greater reductions in body weight, fat mass and BMI across all studies, regardless of procedure type. However, complications were reported in all studies, and ranged from short-term and minor to long-term and severe (requiring reoperation or causing death). Gastric bypass procedures were generally associated with the greatest improvements in weight-related outcomes, but also had the highest complication and mortality rates.

Mental health and quality of life were the primary focus of three SRs [127–129], and the secondary focus of one SR [124] and three RCTs [136–138]. These studies reported improvements in quality of life across treatment groups (sleeve gastrectomy and RYGB [136]; RYGB banded and unbanded [124]) as well as in specific treatment groups (duodenal switch outperforming gastric bypass) [137]. One study [138] also reported improvements in psychosocial functioning following gastric bypass and duodenal switch. One SR [127] (assessing 3 RCTs) reported that bariatric surgery was associated with lower rates and fewer symptoms of mental health conditions. In particular, depression was reduced following bariatric surgery in 11 of 12 studies (two of which were RCTs), while rates of alcohol abuse increased relative to similar populations treated non-operatively. A second SR [128] (assessing 19 RCTs) reported a similar decrease in postoperative depressive symptoms along with improvements in anxiety. However, long-term outcomes were mixed, with some studies reporting an improvement in depressive symptoms lasting upwards of 4 years, while other studies reported an initial postoperative benefit followed by a gradual decline. A third SR and meta-analysis [129] (assessing 21 RCTs) compared mental health outcomes of bariatric surgery using a specific assessment tool (the Short-Form 36). This study reported a significant, consistent and large-magnitude improvement in mental health quality of life following bariatric surgery at 1-year follow-up.

Improvements in eating disorder outcomes were reported in two SRs [128] [127]. One SR [128] (assessing 19 RCTs) reported overall improvements in eating behaviour and body image following bariatric surgery for weight loss in morbidly obese individuals, but noted that not all bariatric surgery patients experienced improvements in mental health. The second SR [127] (assessing three RCTs) reported a reduction in binge eating episodes up to two years post-surgery, followed by an increase at further time points.

### Other approaches

While lifestyle interventions, pharmacotherapy and surgery remain the primary approaches for treating overweight and obesity, one SR [139] and 10 recent RCTs [140–149] have also trialled traditional Chinese medicine, bright light therapy, e-therapies and self-motivation for change through the use of pedometers or self-weighing. In comparison to the other interventions evaluated in this review, the evidence base supporting these interventions were limited (one to two studies in each case). Positive reductions in weight-related outcomes were reported in each intervention, although effect sizes varied. The single RCT [142] assessing a mental health outcome reported improvements in self-efficacy in participants receiving auricular acupressure. None of the included studies reported on eating disorders outcomes or adverse effects.

### Treatment for adolescents

Reduced energy consumption and increased physical activity were the basis for obesity interventions for adolescents, as they were for adults. Pharmacological and surgical interventions were considered in more severe or treatment-resistant cases. Four SRs [150–153] and 13 [154–166] RCTs evaluated weight loss interventions targeting adolescents and reported predominantly positive effects on body weight and related measures. The only trialled intervention that did not reduce weight or a related measure was dance-based exergaming [163]. A number of studies reported significant short-term results that dissipated at longer-term follow-ups. Adverse effects, ranging from mild to severe, were reported in two SRs [150, 153] on bariatric surgery and one RCT [161] prescribing the glucagon-like peptide 1 receptor agonist exanitide to severely obese patients.

One SR and 6 RCTs evaluated mental health and eating disorders outcomes. Quality of life improved following adjustable gastric banding [153] and combined CBT and exercise [160], while psychosocial functioning and body image improved following exercise interventions (cycling exercise [158] and dance based exergaming [163]). Combined diet, exercise and behavioural/psychological interventions led to improvements in depressive symptoms [165], improved body satisfaction and decreased internalization of female norms [157]. One RCT [155] assigned behavioural weight loss plus an anorectic (sibutramine) to ethnically diverse obese adolescents with and without binge eating behaviours, and reported an improvement in cognitive restraint and disinhibition (loss of control over eating) in individuals with and without initial binge eating behaviours.

## Discussion

Numerous treatment strategies exist to assist people in losing weight. Consistent with the literature [58, 167, 168], lifestyle interventions incorporating dietary, exercise and behavioural or psychological components were the most commonly recommended first-line approach, with escalation to pharmacotherapy and bariatric surgery in more severe or treatment-resistant cases. Bariatric surgery and registered medicines consistently reduced weight but were also associated with adverse effects that ranged from mild to severe, while exercise, dietary interventions and behavioural/psychological interventions produced mixed weight loss outcomes but had few adverse effects. Psychological and eating disorder outcomes were infrequently measured (6 of 31 SRs and 31 of 101 RCTs), but where reported, tended to improve alongside weight loss. Specifically, improvements in quality of life, and in some cases depression, were reported in a subset of studies of all intervention types, while improvements in eating disorder symptoms were reported consistently only in interventions incorporating behavioural or psychological treatments. While our review did not explicitly evaluate the factors contributing to psychological improvements, previous studies have implicated weight loss as a key factor in determining changes in psychological state [169–171], with the greatest improvements in psychosocial functioning occurring in individuals experiencing the greatest weight loss [172]. Since body image dissatisfaction, weight-related stigmatization and decreased self-esteem are risk factors for depression [173], it follows that interventions that reduce body weight in obese individuals may also lead to improvements in body satisfaction and other related measures of well-being. Given the reciprocal relationship between obesity and mental health and the benefits of behavioural and psychological interventions on eating disorders outcomes, overweight and obesity interventions that incorporate these components would provide a more balanced approach than the traditional focus on weight loss alone.

Concerns have previously been raised that dieting may precipitate eating disorders in overweight and obese individuals [174], however, our findings suggest that professionally administered weight-loss programmes do not increase the risk or symptoms of eating disorders. In contrast, a large body of evidence [175–177] reports harms from *unhealthy* dieting behaviours, which confer a 5- to 18-fold risk for development of eating disorders. It is important that clinicians are aware of the increased risk for harm in individuals engaging in unhealthy dietary practices, and monitor individuals to ensure that healthy diets do not transition into unhealthy diets.

In Australia, the use of surgical procedures to treat obesity has risen dramatically in recent years [178]. Consistent with recent obesity guidelines (e.g., NHMRC, 2013 [179]) our review found bariatric surgery to be the most effective intervention for sustained weight loss, and the majority of studies that included an assessment of psychological outcomes reported improvements, primarily in quality of life and depression symptoms. However, this benefit is not universal, and approximately 20% of patients undergoing bariatric surgery fail to achieve clinically significant weight loss or experience a worsening in psychological outcomes [128, 180, 181]. For certain individuals who lose large amounts of weight, loose, sagging and excess skin may contribute to worsening body image. For example, one study found that over two thirds of post-bariatric surgery patients viewed the development of excess skin to be a negative outcome of treatment, and for some, a motivator to seek plastic surgery [182]. Further, a growing literature suggests that suicide risk is substantially elevated in patients following bariatric surgery [183–185]. Increased suicidality in this population is strongly associated with depressive symptoms [186], and obese patients who seek surgery have a higher incidence of psychological distress when compared to obese patients who do not seek surgery [187] or seek less invasive forms of treatment [188]. Despite this concerning association, suicidality was not evaluated or reported on in any of the studies included in this review.

A recent Australian study of bariatric surgery candidates reported that 13.5% of individuals met the DSM-IV criteria for BED [189], while subthreshold disordered eating behaviours in the pre-surgical population were anticipated to exceed these rates [190, 191]. Additionally, disordered eating and BED pre-surgery are associated with poor weight loss outcomes, surgical complications and psychological distress [190, 192–194]. For individuals without a pre-existing eating disorder, the development of eating disorders following surgery is an uncommon but serious postoperative issue that requires particular attention by practitioners recommending or performing these procedures [195]. A small body of literature supports the use of behavioural and cognitive approaches for patients who develop symptoms of anorexia nervosa or bulimia nervosa [196–198], but clinicians should be aware of the difficulties they may encounter when treating post-bariatric eating pathologies. These include the challenges of navigating healthy eating behaviours in patients facing postsurgical food intolerance, fear of gaining weight, and intense management of weight and body image concerns [199]. Certain postoperative symptoms may lead patients to engage in restrictive or compensatory behaviours to mitigate discomfort from consumption of foods that are difficult to tolerate post-operatively [200]. For example, post-operative patients may adopt vomiting behaviour after meals to reduce discomfort caused by consuming newly indigestible foods, or as a means of accelerating weight loss [200]. These factors make it challenging to distinguish between normal post-surgery eating behaviours and eating pathology, since many such changes in eating behaviour are necessitated by the surgery and even encouraged by clinicians [195, 201]. In light of these behaviours and the increased vulnerability of the post-surgical population, clinicians should ensure that long-term follow-ups include an assessment of the motivations for abnormal eating behaviours, separating behaviours motivated by physical discomfort from behaviours motivated by weight and shape concerns.

Future research should endeavour to evaluate and develop a more comprehensive metric of treatment success than BMI or body composition measures alone. There is presently a lack of consensus on what an ‘ideal’ BMI is, particularly for individuals losing large amounts of weight [202, 203], and the emphasis that is placed on external measures of health such as BMI may detract from other important measures of health such as physical activity and well-being [204]. The development of standardized tools for eating disorders assessment in the weight loss context and the identification of shared modifiable risk factors for both conditions would contribute to greater identification of disordered eating symptoms and the delivery of more targeted interventions.

### Limitations of this review

The limitations in this review should be taken into account when interpreting outcomes. These include: 1) the RCTs included in evaluated SRs were often highly heterogeneous, precluding pooling for meta-analysis in studies that included these [83]; 2) study outcomes were at times inconsistent [67, 83, 125]; 3) some studies contained a moderate to high risk of bias due to lack of double-blinding and inappropriate or poorly described methods of randomization; and 4) many studies used small, predominantly female and Caucasian samples, limiting generalisability to the broader population. Additionally, this review was limited to a qualitative summary of outcomes reported by original study authors and, by virtue of strict inclusion criteria, may have missed relevant studies that have not yet been published, were published in languages other than English or published prior to 2011. By focusing on uncomplicated obesity, our review may also have missed key studies that explored outcomes in individuals presenting with physical or psychological comorbidities.

## Conclusions and recommendations

There are a number of evidence-based strategies for weight loss, and many of these have the additional benefit of improving quality of life, depression and unhealthy eating behaviours. The weight loss strategy that is most effective will depend on an individual’s unique needs and the characteristics of their illness. These include the severity of their obesity, their treatment history, their history of disordered eating or psychological comorbidities, as well as their willingness to manage adverse effects from treatment. Bariatric surgeries lead to the greatest and most persistent reductions in weight, but are frequently accompanied by adverse effects that range from mild to severe. In contrast, interventions incorporating behavioural or psychological components are less efficacious for weight loss, overall, but cause no adverse effects while leading to improvements in psychological well-being and eating disorders symptomatology. Given the interconnected nature of obesity, mental health and eating disorders, we suggest that weight-loss interventions focus simultaneously on two targets: an evidence-based component that targets weight loss (lifestyle interventions, medications or surgery, dependent on the individual’s circumstance) alongside an evidence-based behavioural or mental health component that focuses on psychological well-being. This second component should include an ongoing evaluation of disordered eating behaviours and psychopathologies.

There is an increased need for prevention and treatment interventions that target the broad spectrum of weight-related disorders. This necessarily requires dialogue and collaboration between professionals in obesity and eating disorders sectors, as well as the involvement of stakeholders at all levels of community and government. Treatment strategies for overweight and obesity should be evidence based and comprehensive, taking into account a broader range of health outcomes than weight or BMI alone [205]. Given the benefits of behavioural and psychological interventions on eating disorders outcomes and weight loss, weight loss interventions should incorporate these components into a more holistic program of care. Treatments should follow a chronic disease model of care that progresses from the use of micro-environmental and lifestyle interventions through to more intensive interventions alongside the severity of obesity [206]. Existing and new treatments should include long-term follow ups and maintenance sessions which incorporate routine assessments of psychological well-being alongside weight-related measures. Importantly, psychological and physical comorbidities should be managed concurrently with weight to maximize outcomes in both physical health and quality of life [207], and when disordered eating is detected, stabilization of eating disorders symptoms should precede and run alongside the weight loss program. This review provides support for ua multidisciplinary treatment approach that takes into account the various factors underlying obesity and eating disorders, as well as the factors confounding treatment outcomes.

## Additional files

Additional file 1: Study assessment procedures: Overview Quality Assessment Questionnaire, Jadad Scale and NHMRC Body of Evidence Matrix. (DOCX 47 kb)

Additional file 2: Table S1. Study features and assessment of risk of bias within individual systematic reviews [32, 33, 45, 46, 52, 61–67, 82, 84–88, 124–132, 139, 150–153, 208] (DOCX 52 kb)

Additional file 3: Table S2. Study features and assessment of risk of bias within individual randomized controlled trials [1, 4, 34–40, 42–44, 47–51, 53–60, 69–76, 78–81, 89–94, 96–109, 111–122, 133–138, 140–149, 154, 155, 157–166, 209–213]. (DOCX 73 kb)

Additional file 4: Table S3. Overview descriptions of interventions included in this review [25, 26, 179, 214–220]. (DOCX 47 kb)

## Abbreviations

BED: Binge eating disorder
BMI: Body mass index
BN: Bulimia nervosa
CBT: Cognitive behavioural therapy
OQAQ: Overview quality assessment questionnaire
RCT: Randomized controlled trial
RYGB: Roux-en-Y gastric bypass
SR: Systematic review
TGA: Therapeutic goods administration
