# Supplementary material for: A systematic review and narrative synthesis of interventions for uncomplicated obesity: weight loss, well-being and impact on eating disorders
Source: J Eat Disord. 2017 May 1;5:15. doi: 10.1186/s40337-017-0143-5 (PMC5410702; doi:10.1186/s40337-017-0143-5)
Supplement: Supplementary file 1 — Study assessment procedures: Overview Quality Assessment Questionnaire, Jadad Scale and NHMRC Body of Evidence Matrix. (DOCX 47 kb) [file 40337_2017_143_MOESM1_ESM.docx]

Additional file 1

| **Assessment of systematic reviews using the Overview Quality Assessment Questionnaire (OQAQ)** |
| --- |
| Were the search methods reported?  Was the search for evidence reasonably comprehensive?  Were the study inclusion criteria reported?  Was selection bias avoided?  Were the criteria for assessing study validity reported?  Was assessment of study validity appropriate?  Were methods to combine studies reported?  Were the findings of studies combined appropriately?  Were the conclusions supported by the data/analysis? |

Scoring: We adapted the scoring of the OQAQ[29] to produce a quality rating of high (all criteria were met), moderate (>5 criteria were met items), or low (<5 criteria were met).

| **Assessment of randomized controlled trials using the Jadad Scale** |
| --- |
| Was the study described as randomized (this includes words such as randomly, random, and randomization)? (+1)  Was the method used to generate the sequence of randomization described and appropriate (table of random numbers, computer-generated, etc)? (+1)  Was the study described as double blind? (+1)  Was the method of double blinding described and appropriate (identical placebo, active placebo, dummy, etc)? (+1)  Was there a description of withdrawals and dropouts? (+1)  Deduct one point if the method used to generate the sequence of randomization was described and it was inappropriate (e.g. patients were allocated alternately, or according to date of birth, hospital number, etc). (+1)  Described but inappropriate (-1)  Deduct one point if the study was described as double blind but the method of blinding was inappropriate (e.g. comparison of tablet vs. injection with no double dummy). (+1)  Described but inappropriate (-1) |

Scoring: We adapted the scoring of the Jadad scale[30] to produce a quality rating of high (score of 5), moderate (score of 3-4) or low (score of 0-2).

**Evidence summary procedure *(*NHMRC Body of Evidence Matrix)**

|  | **Excellent** | **Good** | **Satisfactory** | **Poor** |
| --- | --- | --- | --- | --- |
| **Evidence base** | Several high quality SRs/RCTs with low risk of bias | 1-2 high quality SRs/RCTs studies with low risk of bias | SRs/RCTs with moderate quality and/or moderate risk of bias | SRs/RCTs with low quality and/or high risk of bias |
| **Consistency** | All studies consistent | Most studies consistent and inconsistency accounted for | Some inconsistency, reflecting genuine uncertainty around question | Inconsistent evidence |
| **Clinical impact** | Very large | Moderate | Slight | Restricted |

The evidence summary procedure was adapted from the NHMRC *Levels of evidence and grades for recommendations for developers and guidelines*[31]*.* SR= Systematic review; RCT= randomized controlled trial. When only a single RCT was available for an intervention, consistency was scored as N/A. When a single SR was available, consistency was scored based on the consistency between RCTs evaluated in the SR.
