# Supplementary material for: A systematic review and narrative synthesis of interventions for uncomplicated obesity: weight loss, well-being and impact on eating disorders
Source: J Eat Disord. 2017 May 1;5:15. doi: 10.1186/s40337-017-0143-5 (PMC5410702; doi:10.1186/s40337-017-0143-5)
Supplement: Supplementary file 2 — Study features and assessment of risk of bias within individual systematic reviews [32, 33, 45, 46, 52, 61–67, 82, 84–88, 124–132, 139, 150–153, 208] (DOCX 52 kb) [file 40337_2017_143_MOESM2_ESM.docx]

**Additional file 2**

| **Author, year,**  ***N* RCTs, demographic** | **Intervention(s) reviewed** | **Outcomes and clinical impact** | **Adverse effects** | **Risk of bias** |
| --- | --- | --- | --- | --- |
| **Astell**, **2013**[82], *N*=14, obese adults | Anorectic plant extracts | + WL outcomes were largely inconclusive | ++ | ++ |
| **Baillott**, **2015**[33], *N*=7, obese adults | Physical activity, exercise, counselling and education | ++ Sig improvements in fat and waist circumference; long-term TXs had better effects than short- and intermediate-term; NS impact on QOL | N/A | ++ |
| **Baillott, 2014**[52], *N*=8, obese adults | Physical activity or exercise | ++ Diet/exercise is more effective than diet alone in WL | N/A | + |
| **Barnes**, **2015**[67], *N*=24, overweight/ obese adults | Motivational interviewing | + Inconsistent evidence for impact on WL | N/A | ++ |
| **Black, 2013**[150], *N*=1; obese youth 6-18yrs | Bariatric surgery | +TX led to sig, short-term WL (only 1 RCT with high attrition) | ++ | + |
| **Booth, 2014**[61], *N*=15, overweight/ obese adults | BWL Txs (diet + exercise) | + TX led to minimal WL | N/A | ++ |
| **Bouza, 2012**[152], *N*=9, overweight/ obese adolescents | Antidiabetic medication (metformin) | ++ Combined with lifestyle interventions, medication was effective in reducing BMI | N/A | ++ |
| **Brufani, 2014**[151], *N*=9, obese children/ adolescents | Antidiabetic medication (metformin); lifestyle interventions | ++ Small but sig WL over the short-term | N/A | ++ |
| **Buchwald, 2014**[124], *N*=3, obese adults | Surgery (banded and non-banded RYGB) | +++RYGB led to sig intermediate-term WL, and banded outperformed non-banded; surgery (both banded/ non-banded) did not adversely affect QOL | ++ | ++ |
| **Chang, 2014**[125], *N*=37, obese adults | Surgery (gastric bypass, AGB, VBG) vs non-surgery comparators | +++ Surgery led to sig long-term WL | +++ | ++ |
| **Colquitt, 2014**[126], *N*=7, overweight/ obese adults | Bariatric surgeries vs non-surgery comparators | ++ Surgery led to sig BMI reductions that were higher in individuals with higher BMIs (≥40)  ++ Surgery sig improved QOL compared with non-surgical TXs | ++ | + |
| **Dawes, 2015**[127], *N*=5, overweight/ obese adults | Bariatric surgeries | ++Surgery was strongly associated with reductions in depression and depressive symptoms | N/A | + |
| **Gloy, 2013**[131], *N*=11, obese adults | Bariatric surgeries vs non-surgery comparators | ++ Surgery sig improved BMI compared with non-surgical TXs; improvement was greatest for those with BMI>40  ++Surgery sig improved QOL compared with non-surgical TXs | ++ | + |
| **Gudzune, 2015**[32], *N*=39, overweight/ obese adults | Commercial WL programs, education or behavioural counselling | +++ The 3 top programs produced sig and consistent WL over the longer-term | + | ++ |
| **Hutchesson, 2015**[139], *N*=84, overweight/ obese adults | eTherapies | ++ TXs incorporating evidence-based features led to sig greater WL | N/A | ++ |
| **Hutchesson, 2013**[63], *N*=8, overweight/ obese women 18-35yrs | BWL and behavioural weight management | ++TX sig reduced weight compared with control in most (5/8) studies | N/A | ++ |
| **Johns, 2014**[64], *N*=8, overweight/ obese adults | Behavioural weight management, diet, physical activity | ++ Behavioural weight management combined with physical activity or diet compared with either activity or diet alone led to sig WL | N/A | ++ |
| **Jurgens, 2012**[208], *N*=14, overweight/ obese adults | Green tea | Green tea had an NS impact on WL | ++ | ++ |

| **Author, year,**  ***N* RCTs, demographic** | **Intervention(s) reviewed** | **Outcomes and clinical impact** | **Adverse effects** | **Risk of bias** |
| --- | --- | --- | --- | --- |
| **Kaiser, 2014**[46], *N*=7; not reported | Increased fruit and vegetable intake | TX had a clinically NS impact on WL | N/A | ++ |
| **Kubik, 2013**[128], *N*=9; not reported | Bariatric surgery | ++Surgery improved psychopathology, depressive symptoms and QOL in *some* patients; +surgery improved eating behaviour and body image in *some* patients | N/A | +++ |
| **Magallares, 2015**[129], *N*=21; not reported | Bariatric surgery | +++Physical health was sig improved post-surgery group compared with pre-surgery; +++QOL was sig improved post-surgery group compared with pre-surgery | N/A | +++ |
| **Naude, 2012**[45], *N*=14; overweight/ obese adults | Low-carb and balanced diets | +Low-carb and balanced diets produce similar WL; energy reduction rather than altered macronutrients lead to WL | N/A | ++ |
| **Nigro, 2013**[88], *N*=4, not reported | Anti-obesity medication (Lorcaserin) | ++Medication led to sig WL compared with placebo | ++ | +++ |
| **Olson, 2015**[66], *N*=8, overweight/ obese adults | Mindfulness-based interventions | ++Mindfulness-based TXs led to sig WL compared with control TXs | N/A | ++ |
| **Onakpoya, 2014**[85], *N*=9, overweight/ obese individuals | Chromium supplements | +TX sig reduced weight and percent body fat, but not BMI or waist to hip ratio | + | + |
| **Onakpoya, 2013**[84]**,** *N*=20, overweight/ obese individuals | Glucomannan supplements | No sig TX effects | ++ | + |
| **Pathak, 2014**[86], *N*=18, overweight/ obese women | Vitamin D | +No effect of vitamin D on body weight or fat in the absence of caloric restriction | N/A | ++ |
| **Puzziferi, 2014**[130], *N*=10, severely obese adults | AGB, RYGB | +++RYGB led to substantial and sustained (2-5yrs) WL that was sig greater than WL from AGB | +++ | ++ |
| **Trastulli, 2013**[132], *N*=15, obese adults | SG | ++SG led to sig WL that was similar to alternative surgical procedures | ++ | ++ |
| **Wadden, 2015**[65], *N*=12, overweight/ obese individuals | BWL and lifestyle interventions | + Some evidence for WL | N/A | +++ |
| **Willcox, 2014**[153], *N*=1, overweight/ obese adolescents | AGB | +++AGB sig reduced BMI  AGB sig improved QOL | ++ | ++ |
| **Yanovski, 2014**[87], *N*=20, obese adults | Anti-obesity medications (orlistat, lorcaserin, phentermine + topiramate) | +++All medications used alongside lifestyle interventions increased the probability of clinically meaningful WL | ++ | +++ |
| **Yoong, 2013**[62], *N*=16, overweight/ obese adults | BWL delivered by primary care physicians | +High-intensity WL counselling delivered by primary-care physicians did not lead to sig WL | N/A | +++ |

**Notes**: Only the number of RCTs is given but SRs may have included additional, non-RCT studies. The following conventions apply to all terms: Low: +, Moderate: ++, High: +++. Outcomes reflect the clinical impact of the study’s findings for physical, psychological and eating disorder (ED) outcomes. Clinical impact was ranked as low, moderate or high after assessing the statistical precision, effect size, clinical relevance and duration of impact for each study/outcome. Risk of bias was evaluated for each study using the 9-point Overview Quality Assessment Questionnaire (OQAQ). **Abbreviations**: BMI: body mass index; N/A: not assessed; NS: non-significant; QOL: quality of life; Sig: statistically significant; Tx: treatment; WL: weight loss. **Interventions**: BWL: behavioural weight loss; AGB: laparoscopic adjustable gastric banding; SG: laparoscopic sleeve gastrectomy; RYGB: Roux-en-Y gastric bypass; VBG: vertical banded gastroplasty
