# Supplementary material for: A systematic review and narrative synthesis of interventions for uncomplicated obesity: weight loss, well-being and impact on eating disorders
Source: J Eat Disord. 2017 May 1;5:15. doi: 10.1186/s40337-017-0143-5 (PMC5410702; doi:10.1186/s40337-017-0143-5)
Supplement: Supplementary file 3 — Study features and assessment of risk of bias within individual randomized controlled trials [1, 4, 34–40, 42–44, 47–51, 53–60, 69–76, 78–81, 89–94, 96–109, 111–122, 133–138, 140–149, 154, 155, 157–166, 209–213]. (DOCX 73 kb) [file 40337_2017_143_MOESM3_ESM.docx]

Additional file 3

| **Author, year, *N*, demographic, setting, follow-up** | **Intervention(s) reviewed** | **Outcomes and clinical impact** | **Adverse effects** | **Risk of bias** |
| --- | --- | --- | --- | --- |
| **Allison, 2011**[90]**,** *N*=1267 obese adults; 56 week follow-up | 1. Phentermine 3.75mg + topiramate 23mg; 2. Phentermine 15mg + topiramate 92mg; 3. Placebo pill | +++Greater WL from TX 1and2 than control; statistically and clinically greater WL in TX1 than TX2 | + More common in TX 2 | + |
| **Almeida, 2015**[42]**,** *N*=1001 overweight/ obese adults; 6 month follow-up | 1.Internet-based INCENT WL support Program; 2.Control: Email-based Livin’ My Weigh control program | +Minimal WL in both groups; NS group differences | N/A | ++ |
| **Anderson, 2011**[34]**,** *N*=22 overweight adults; 24 week follow-up | 1. BWL (diet + exercise); 2. Usual-care counselling | ++ Greater WL from TX compared to control at 8, 16 and 24 week follow-ups | N/A | ++ |
| **Apovian, 2013**[118]**,** *N*=1001 overweight/ obese adults; 28 and 56 week follow-up | 1. Naltrexone 62mg +bupropion 360mg; 2. Placebo pill | +++Greater WL from TX compared to control at 28 and 56 weeks; TX group sig improved in physical function, self-esteem and sexual life | ++ | + |
| **Aronne, 2013**[91]**,** *N*=756; 28 week follow-up | 1. Phentermine 7.5mg; 2. Topiramate ER 46mg; 3. Phentermine/Topiramate ER 7.5mg/46mg; 4. Phentermine 15mg; 5. Topiramate ER 92mg; 6. Phentermine/Topiramate ER 15/92mg; 7.Placebo pill control. All participants received lifestyle intervention counselling | +++ TX 3 and TX 6 were the most effective for WL, and led to greater improvements than all other TXs or placebo | + | + |
| **Baer, 2011**[92]**,** *N*=90 overweight/ obese adults; 23 week follow-up | 1. Whey Protein supplement; 2. Soy Protein supplement; 3. Isoenergetic amount of carbohydrate (control) | ++Greater WL from TX 1 than control (small effect size); NS differences between TX 2 and control or TX 1 and 2 | N/A | + |
| **Barnes, 2014**[81]**,** *N*=89 overweight/ obese adults; 3 month follow-up | 1. Motivational interviewing, 5 sessions over 12 weeks; 2. Nutrition psychoeducation, 5 sessions over 12 weeks; 3. Usual care control | ++Greater WL and reduction in depression and BED symptoms from TX compared with control; NS difference in WL between TX 1 and 2 and between participants with and without BED | N/A | ++ |
| **Bays, 2014**[93]**,** *N*=376 overweight/ obese adults; 12week follow-up | 1. Canagliflozin 50mg; 2. Canagliflozin 100mg; Canagliflozin 300mg; Placebo control | +Greater WL in all TX groups compared with control (small effect size) | ++ | + |
| **Benito, 2015**[209]**,** *N*=120 overweight/ obese adults | 1. Strength training; 2. Endurance training, running, cycling or optional activities; 3. Strength + endurance training; 4. General physical activity advice. All groups received a hypocaloric diet. | +++All forms of exercise resulted in statistically and clinically sig weight loss, with NS differences between groups | N/A | ++ |
| **Berkowitz, 2011**[154]**,** *N*=113 overweight/ obese adolescents; 4 and 12 month follow-up | 1. Meal replacement SlimFast shake consumed TD; 2. Conventional diet of 1,300 – 1,500kcal/day | ++TX 1 produced statistically and clinically sig reductions in BMI after 4 but not 12 months compared to TX2 | N/A | ++ |
| **Bishop-Gilyard, 2011**[155]**,** *N*=82 overweight/ obese adolescents; 1, 6 and 12 month follow-up | 1. Sibutramine 15mg/day + behavioural counselling; 2. Placebo pill + behavioural counselling | +++Greater BMI reduction in TX 1 than control at 12 months; NS differences between participants with and without BED; sig reductions in BE in both groups but NS differences between groups | N/A | ++ |
| **Blomquist**[89]**, 2011,** *N*=82 obese adults; 3 month follow-up | 1. Orlistat 120mg, 3 x day + 12 weeks of CBT; 2. Placebo pill control +12 weeks of CBT | ++Sig reduction in WL and BE symptoms in TX 1 compared to control at 12 weeks | + TX 1 | ++ |
| **Brennan, 2013**[210]**,** *N*=63 overweight/ obese adolescents; 6 month follow-up | 1. CBT + motivational interviewing + diet + exercise advice; 2. No TX control | +Greater WL in TX 1 than control group (but small effect size) | N/A | ++ |
| **Author, year, *N*, demographic, setting, follow-up** | **Intervention(s) reviewed** | **Outcomes and clinical impact** | **Adverse effects** | **Risk of bias** |
| **Burke, 2011**[211]**,** *N*=210 overweight/ obese adults**;** 6- month follow-up | 1. Personal Digital Assistant; 2. PDA with feedback; 3. Paper record control | ++Sig WL in all groups at 6 months; NS difference between groups | N/A | +++ |
| **Cakmakci, 2011**[60]**,** *N*=63 overweight/ obese adults; 8week follow-up | 1. Pilates sessions (1 hour 4x/week); 2. No TX control | NS difference between groups | N/A | +++ |
| **Carraca, 2012**[35]**,** *N*=225 overweight/ obese women; 24 month follow-up | 1. Weight management intervention + exercise; 2. General health education control | ++Sig WL in TX 1 compared to the control group; Sig improvements in body image, but NS differences between groups | N/A | +++ |
| **Cayir**, **2015**[145], *N*=40 obese women; 3 month follow-up | 1. Pedometer + low calorie diet + exercise prescription; 2. No pedometer + low calorie diet + exercise prescription | +++Greater WL in TX 1 compared to controls | N/A | ++ |
| **Cesa**, **2013**[69], *N*=90 obese women; 1 year follow-up | 1. Enhanced CBT + virtual reality protocol; 2. Standard CBT; 3.Inpatient TX control | ++Sig WL in both groups; NS difference between groups | N/A | + |
| **Chambliss**, **2011**[147], *N*=120 overweight adults | 1. Computerised self-monitoring with basic feedback; 2. Computerised self-monitoring with enhanced behavioural feedback. 3. No TX waitlist control | ++Sig WL in TX 1 and TX 2 after 12 weeks compared to controls; NS differences between TX groups | N/A | ++ |
| **Christensen**, **2011**[36], *N*=98 overweight female health care workers; 3 month follow-up | 1. Calorie restricted diet plan + strengthening exercise + CBT; 2. Monthly oral lecture control | ++Greater reductions in weight, fat, and waist circumference in TX 1 compared with controls | N/A | ++ |
| **Danilenka**, **2013**[140], *N*=39 overweight adult women, 3 and 10 week follow-up | 1. 3 weeks of daily bright light TX; 2. Placebo using imitation device | +Greater reduction in body fat in TX 1 than control; NS difference in WL between groups | N/A | ++ |
| **DeBar**, **2012**[157], *N*=208 adolescent females, 6 and 12 month follow-up | 1. Multi-component lifestyle intervention including dietary advice, exercise promotion + counselling; 2. Usual primary care control | ++Greater reductions in BMI and psychological outcomes (improved body satisfaction and decreased internalization of female norms) in TX 1 compared with control | N/A | ++ |
| **DeFina**, **2011**[94], *N*=128 overweight/ obese adults; 6 month follow-up | 1. Omega-3 supplements + calorie controlled diet + exercise prescription; 2. Placebo pill + calorie controlled diet + exercise prescription | +NS difference in WL between groups; all participants lost more than 5% of their body weight | N/A | +++ |
| **Donnelly**, **2013**[54], *N*=141 overweight/ obese adults; 10 month follow-up | Treatment: Aerobic exercise 5x/week for 10 months. 1. Targets 400kcal reduction/session in males; 2. Targets 400kcal reduction/session in females; 3. 600kcal reduction in males; 4. Targets 600kcal reduction/session in females; 5. No exercise in males; 6. No exercise in females | +++Sig reductions in weight and body fat in TX 1-4 compared with controls; NS differences between TX groups; NS differences between males and females | N/A | ++ |
| **Forman**, **2013**[70], *N*=128 overweight/ obese adults; 6 month follow-up | 1. Acceptance-based behavioural therapy weekly for 40 weeks; 2. Standard behavioural therapy weekly for 40 weeks | ++Sig improvements in weight and QOL in both groups at 6 months; NS differences between groups | N/A | ++ |
| **Foster**, **2012**[37], *N*=123 overweight/ obese adults; 6 and 18 month follow-up | 1. Hypocaloric almond-enriched diet + behavioural weight control; 2. Hypocaloric nut free diet + behavioural weight control | ++Greater WL in TX 1 than control at 6- but not 18-month follow-up | N/A | ++ |

| **Author, year, *N*, demographic, setting, follow-up** | **Intervention(s) reviewed** | **Outcomes and clinical impact** | **Adverse effects** | **Risk of bias** | |
| --- | --- | --- | --- | --- | --- |
| **Gadde**, **2011**[212], *N*=225 obese adults; 12 month follow-up | 1. 200mg zonisamide/day + diet and lifestyle counselling; 2. 400mg zonisamide/day + diet and lifestyle counselling; 3. Placebo pill + diet and lifestyle counselling | ++Greater WL in TX 2 compared with TX 1 or control at 12-month follow-up; NS differences in WL between TX 1 and control | ++ TX 1 and 2 | + |  |
| **Georg**, **2012**[96]; *N*=96 obese adults; 12 week follow-up | 1. Alginate supplement + calorie restricted diet; 2. Placebo supplement + calorie restricted diet | ++Greater WL in TX 1 than control in the completer analysis but not ITT analysis | N/A | ++ |  |
| **Goldfield**, **2012**[158]; *N*=30 overweight/ obese adolescents; 10 week follow-up | 1. Cycling as part of a video game 2x/week for 10 weeks; Cycling to music 2x/week for 10 weeks | ++Both groups had improvements in social competence and body image; NS difference between group in body composition or weight | N/A | ++ |  |
| **Gorin, 2013**[38]**;** *N*=201 overweight/ obese adults | 1. BWL + calorie and fat restricted diet + changes to home environment; 2. BWL loss + calorie and fat restricted diet | ++Greater WL in TX 1 women at 6- but not 18-month follow-up compared to controls; NS effect for men in either group | + | +++ |  |
| **Griffin, 2013**[47]**;** *N*=71 overweight young women; 6 and 12 month follow-up | 1. High protein diet; 2. High carbohydrate diet | ++Greater WL and % fat loss in TX 1Tx compared to controls at 6- but not 12- month follow-up | N/A | +++ |  |
| **Grilo, 2014**[101]**,** *N*=48 obese adults with BED**;** 6 and 12 month follow-up | 1. Self-help CBT for 4 months; 2. Sibutramine 15mg/day for 4 months; 3. CBT + sibutramine15mg/day for 4 months; 4. CBT + placebo for 4 months | ++ Greater WL in TX 2 compared with other groups at 4- but not 6- or 12-month follow-up; reductions in depression in all groups but NS differences between groups; NS differences in BED remission rates at 6- and 12-month follow-up | N/A | + |  |
| **Grilo, 2013**[72]**,** *N*=48 obese adults with BED**;** 4 month follow-up | 1. Self-help CBT over 16 weeks; 2. Usual care over 16 weeks | ++NS reductions in BMI in both groups; sig reductions in BE, ED psychopathology and depressive symptoms but NS differences between groups | N/A | + |  |
| **Grilo, 2013**[100]**,** *N*=79 obese adults with BED; 6 month follow-up | 1. Orlistat +BWL (BED); 2. Orlistat + BWL (non-BED); 3. BWL + placebo (BED); 4.Orlistat + placebo (non-BED) | ++Sig WL in obese individuals without BED but not with BED; NS effect of orlistat added to BWL on psychological outcomes | + Orlistat | ++ |  |
| **Grilo, 2012**[99]**,** *N*=81 overweight/ obese adults with BED; 6 and 12-month follow-up | 1. Fluoxetine; 2. Fluoxetine + CBT; 3. CBT + placebo | ++NS effect of any TX on WL; NS group difference in BED remission rates at 6-months, but sig differences at 12-months (remission in 4%, 27% and 36% in TX 1, 2 and 3, respectively) | N/A | + |  |
| **Grilo, 2011**[71]**,** *N*=125 obese adults with BED**;** 6 and 12 month follow-up | 1. CBT; 2. BWL; 3. CBT + BWL. All TXs were administered via 16 60min sessions over 24 weeks | ++All groups had clinically sig BED remission but group differences were NS; TX 1 had greater reductions in BE than TX 2; TX 2 and 3 had greater WL than TX 1 at 6 months; NS group differences in WL at 12 months | N/A | + |  |
| **Grube, 2012**[97]**,** *N*=125 overweight/ obese adults; 12 week follow-up | 1. Litramine supplement + diet/ exercise advice; 2. Placebo pill + diet/exercise advice | +++Greater reductions in weight, BMI, body fat and waist circumference in TX 1 compared to control at 12-week follow-up | N/A | + |  |
| **Harden, 2014**[98]**,** *N*=40 overweight/ obese women; 12 week follow-up | 1. Docosahexaenoic supplement; 2. Oleic Acid | +Sig WL in both groups; NS difference between groups | N/A | + |  |
| **Hedberg, 2012**[134]**,** *N*=47 obese adults; 4yr follow-up | 1. Duoedenal switch; 2. RYGB | +++Both TXs resulted in statistically and clinically sig reduction in BMI at 4 years; greater WL in TX 1 compared with TX 2 in patients with BMI > 48kg/m^2^ | +++ | ++ |  |
| **Hofsteeng, 2014**[159]**,** *N*=122 obese adolescents**;** 18 month follow-up | 1. Go4it lifestyle intervention that includes dietary and exercise advice; 2. Usual care control | ++Greater reductions in BMI in TX 1 than control; greater reductions for non-Western participants | N/A | ++ |  |

| **Author, year, *N*, demographic, setting, follow-up** | **Intervention(s) reviewed** | **Outcomes and clinical impact** | **Adverse effects** | **Risk of bias** | |
| --- | --- | --- | --- | --- | --- |
| **Hofsteeng, 2013**[160]**,** *N*=122 obese adolescents; 6 month follow-up | 1. Go4it lifestyle intervention that includes dietary and exercise advice; 2. Usual care control | ++Small but sig benefits from TX compared to control in physical health and QOL | N/A | ++ |  |
| **Hsieh, 2011**[141]**,** *N*=55 overweight Asian young adults; 8 week follow-up | 1. Acupressure with Japanese Magnetic Pearls; 2. Placebo control | +++Greater reduction in weight & waist circumference from TX than control | N/A | +++ |  |
| **Huerta, 2015**[115]**,** *N*=97 overweight/ obese women; 10 week follow-up | 1. Eicosapentaenoic acid + energy restricted diet; 2. Alpha-lipoic acid + energy restricted diet; 3. Eicosapentaenoic acid + Alpha-lipoic acid + energy restricted diet; 4. Placebo + energy restricted diet | +++All groups had sig reductions in weight, waist and hip circumference, and waist to hip ratio; greater reductions in weight and waist and hip circumference in TX 2 and 3 compared to TX 1 and control | N/A | ++ |  |
| **Hunt, 2014**[73]**,** *N*=747 overweight/ obese men**;** 12 month follow-up | 1. WL program that included dietary and exercise advice; 2. Wait list control | ++Greater reduction in weight, waist circumference and percentage of body fat in TX 1 compared to wait-list control | + TX | ++ |  |
| **Jakicic, 2012**[74]**,** *N*=363 overweight/ obese adults**;** 18 month follow-up | 1. Stepped-care WL intervention that included a low calorie diet + weekly to monthly counselling sessions; 2. Standard BWL | ++Sig WL in both groups at 18-month follow-up | N/A | ++ |  |
| **Johnston, 2013**[75]**,** *N*=292 overweight/ obese adults**;** 3 and 6 month follow-up | 1. Weight watchers programme that included a food and exercise plan, skills to change behaviour and access to group support; 2. Self-help WL control | +++Greater WL at 3 and 6 months in TX 1 compared controls; Greater WL in participants in TX 1 who used all 3 WL components than those who used 1 or 2 components | N/A | ++ |  |
| **Jung, 2014**[102]**,** *N*=54 overweight/ obese adults; 10 week follow-up | 1. Yeast hydrolysate supplement taken daily; 2. Placebo pill control | +++ Greater reductions in weight, body fat mass and abdominal fat mass at 10 weeks in TX 1 compared to placebo pill controls | N/A | ++ |  |
| **Keating, 2014**[53]**,** *N*=39 overweight adults; 12 week follow-up | 1. High intensity interval training for 20–24 minutes, 3x/week; 2. Continuous aerobic exercise training for 36–48 minutes, 3x/week; 3. Placebo exercise control condition (included stretching, using a fit ball and self-massage) | + Greatest reduction in body fat in TX 2; no sig changes in body mass in any group | + | ++ |  |
| **Kehagias, 2011**[133]**,** *N*=60 morbidly obese adults; 2 and 3yr follow-up | 1. SG; 2. RYGB | ++ Both procedures led to sig WL; greater WL from TX 1 than TX 2 at 2- but not 3-year follow-up | +++ | + |  |
| **Keithley, 2013**[103]**,** *N*=53 overweight/ obese adults; 8 week follow-up | 1. Glucomannan supplement; 2. Placebo pill control | NS effect of either TX on WL and body composition at 8 weeks | + | + |  |
| **Kelly, 2013**[161]**,** *N*=26 severely obese adolescents; 3 month follow-up | 1. Glucagon-like peptide-1 receptor agonist; 2. Placebo pill control | ++Greater WL and BMI reduction in TX 1 compared to placebo pill control (modest effect size) | ++ | ++ |  |
| **Khoo, 2014**[48]**,** *N*=48 obese Asian males; 40 week follow-up | 1. Optifast meal replacements, 2 sachets/ day; 2. Conventional reduced-fat diet | ++ Sig WL and QOL improvements in both groups at 40 weeks; greater reduction in wait circumference and body fat in TX 1 compared with controls | N/A | +++ |  |
| **Kim, 2014**[142]**,** *N*=58 overweight/ obese female college students; 4 week follow-up | 1. Auricular acupressure using Sinapsis alba seeds; 2. Placebo pill control | ++Greater WL and reduction in BMI in TX 1 compared with control; NS group difference in percentage of body fat or waist-to-hip ratio; self-efficacy improved sig for TX 1 but decreased for controls | N/A | +++ |  |
| **Kong, 2014**[162]**,** *N*=104 obese adolescents; 6 month follow-up | 1. Low glycaemic index diet + fortnightly dietary counselling; 2. conventional Chinese diet | ++ Greater reduction in BMI, body weight and waist circumference in TX 1 compared with controls | N/A | ++ |  |

| **Author, year, *N*, demographic, setting, follow-up** | **Intervention(s) reviewed** | **Outcomes and clinical impact** | **Adverse effects** | **Risk of bias** | |
| --- | --- | --- | --- | --- | --- |
| **Kreider, 2011**[213]**,** *N*=90 overweight/ obese sedentary women; 10 and 34 week follow-up | 1. Meal replacement (Special K) + encouragement to increase exercise; 2. Structured meal plan diet + supervised exercise regime | +++Greater WL at 10 and 34 weeks in TX 2 than TX 1, although both groups experienced sig WL | N/A | +++ |  |
| **Lim, 2011**[116]**,** *N*=203 overweight/ obese young women; 12 week follow-up | 1. Metformin + lifestyle intervention; 2. Lifestyle intervention; 3. Placebo + lifestyle intervention control | ++ Greater reductions in weight and waist circumference in TX 2 than TX 3 and control | ++ | + |  |
| **Liu, 2013**[122]**,** *N*=90 overweight/ obese adults**;** 24 week follow-up | 1. 200mg caffeine/20mg ephedrine, 3x/day; 2. Leptin daily; 3. Caffeine/ ephedrine + leptin daily | ++ TX 1 and 3, but not 2, led to sig greater WL at 24 week follow-up; this was attributed to sig greater reductions in overall fat mass | N/A | ++ |  |
| **Maddison, 2011**[44]**,** *N*=322 overweight/ obese children; 24 week follow-up | 1. Active video game, 60 minutes every day of the week; 2. Sedentary video game, playing times left to the discretion of the participants | +Greater reductions in body weight and percentage of overall body fat in TX 1 compared with TX 2 (small effect size) | N/A | ++ |  |
| **Malkina-Pykh, 2012**[39]**,** *N*=58 overweight/ obese adults**;** 12 week follow-up | 1. Rhythmic movement therapy; 2. CBT; 3. No treatment | +++ Greater reduction in BMI, body dissatisfaction, restrained and emotional eating and emotional dysfunction in TX 1 compared with TX 2 and control | N/A | +++ |  |
| **Mangine, 2012**[104]**,** *N*=50 overweight/ obese adults; 4 and 8 week follow-up | 1. 40mg N-oleyl- phosphatidylethanolamine + 35mg epigallocatechin-3-gallate, 3x/day; 2. Placebo pill, 3x /day | +NS effects of TX 1 on WL; a small but sig effect of TX 1 on tension | ++ | ++ |  |
| **Masheb, 2011**[40]**,** *N*=50 obese adults with BED; 6 and 12 month follow-up | 1. CBT + low energy density diet; 2. CBT + general nutrition counselling | ++NS differences between groups at 6 and 12 months, but 26% of all participants achieved greater than 5% WL at 6 months and 30% of participants achieved similar WL at 12; both groups had sig improvement in the behavioural and attitudinal features of BED | N/A | ++ |  |
| **Matthews, 2012**[49]**,** *N*=70 overweight adults; 2, 4 and 6 week follow-up | 1. Ready to eat Kellogg’s breakfast cereal; 2. Usual evening snack | + NS differences in WL between groups; greater reduction in waist circumference for at 6 weeks for TX 1 compared with control | N/A | +++ |  |
| **Moreno, 2014**[50]**,** *N*=79 obese adults; 12 month follow-up | 1. Very low-calorie-ketogenic diet as part of a commercial WL program - Pronokal Method; 2. Standard low calorie diet (caloric value of 10% below the total metabolic expenditure of each individual) | +++ Greater WL in TX 1 at all monthly evaluations; the maximum WL was observed at 8 months; TX 1 led to WL of more than 10% in body weight in 96% of its participants, compared to only 3% of TX 2 participants |  | +++ |  |
| **Munro, 2013**[105]**,** *N*=35 obese adults; 12 week follow-up | 1. Fatty acid supplement + low energy diet; 2. Placebo + low energy diet | ++ Both groups had sig WL at 12 weeks, but the difference between groups was NS | N/A | ++ |  |
| **Nackers, 2013**[41]**,** *N*=125 obese women; 6 and 12 month follow-up | 1. 1,000 calorie diet + behavioural treatment; 2. 1,500 calorie diet + behavioural treatment | +++ Both TX groups had sig WL at 6- and 12-month follow-up; TX 1 led to sig more weight re-gain between 7-12 months; NS difference in WL at 12-month follow-up | N/A | +++ |  |
| **Nanchahal, 2012**[76]**,** *N*=381 overweight/ obese adults; 12 month follow-up | 1. 1 to 1 BWL program that included dietary and exercise advice; 2. Usual care control | +++ TX 1 resulted in a sig greater reduction in weight and waist circumference than usual care control | N/A | ++ |  |
| **Napolitano, 2013**[144]**,** *N*=52 overweight/ obese students; 4 and 8 week follow-up | 1. WL interventions accessed through Facebook; 2. Similar to TX 1 with inclusion of targeted support via text messaging; 3. Wait-list control | ++ Greater WL at t 4 weeks in TX 1 than TX 2 or wait-list control | N/A | +++ |  |
| **Park, 2014**[107]**,** *N*=80 obese adults; 12 week follow-up | 1. *Gynostemma pentaphyllum* extract 450mg/day; 2.Placebo pill, taken once daily | + Greater reductions in abdominal fat and body weight in TX 1 compared to controls (small effect size) | N/A | +++ |  |
| **Author, year, *N*, demographic, setting, follow-up** | **Intervention(s) reviewed** | **Outcomes and clinical impact** | **Adverse effects** | **Risk of bias** | |
| **Pataky, 2013**[106]**,** *N*=289 obese adults with BED; 6 month follow-up | 1. Rimonabant 20mg/day; Placebo pill, daily | +++ Greater reduction in weight, waist circumference and binge eating in TX 1 compared with controls | ++ | ++ |  |
| **Pellegrini, 2012**[78], *N*=51 overweight/ obese adults; 6 month follow-up | 1. Technology-based WL; 2. Standard BWL + a technology based element; 3. Standard BWL | ++ All TXs resulted in sig reductions in body weight, wait circumference, hip circumference and percentage of body fat; NS differences between groups | N/A | +++ |  |
| **Pinto, 2013**[79], *N*=144 overweight/ obese adults; 24 and 48 week follow-up | 1. Standard BWL; 2. BWL + weight watchers program; 3. Weight watchers program | ++All TXs resulted in sig WL at 12, 24 and 48 weeks; NS differences between groups | N/A | +++ |  |
| **Pi-Sunyer, 2015**[108], *N*=3731 obese adults; 56 week follow-up | 1. 3mg Liraglutide injection, once daily + lifestyle modification counselling; 2. Placebo injection + lifestyle modification counselling | +++ Greater reduction in weight and waist circumference at 56 weeks in TX 1 compared to the placebo group | ++ | + |  |
| **Poelman, 2015**[51], *N*=278 overweight and obese adults | 1. PortionControl@Home program that aims to modify dietary behaviours; Wait list control | ++ Greater reductions in BMI at 3- but not 12-months in TX 1 compared to the control group | N/A | ++ |  |
| **Poole, 2011**[109], *N*=24 overweight college students; 8 week follow-up | 1. WL supplement + exercise; 2. Placebo pill + exercise | + NS differences in BMI or body composition between groups; NS effect of TX | N/A | ++ |  |
| **Reichkendl, 2014**[58], *N*=64 overweight Caucasian men; 11 week follow-up | 1. Moderate exercise, 3x/ week; 2. High intensity of exercise, 3x/ week; 3. No exercise | +++Greater reductions in weight and waist circumference and improvements in QOL in TX 1 and TX 2 compared with no exercise controls | N/A | +++ |  |
| **Ross, 2015**[59], *N*=300 abdominally obese adults; 24 week follow-up | 1. Low amount, low intensity of exercise 5x/week; 2. High amount, low intensity of exercise 5x/week; 3. High amount, high intensity 5x/week; 4. No prescribed exercise control | ++ All TX groups had sig reductions in body weight and waist circumference compared to controls at 24 weeks | N/A | ++ |  |
| **Sakurai, 2013**[149], *N*=66 overweight older adults; 3 month follow-up | 1. Dietary modification (4x 75-min nutrition guidance classes) + 75 minutes of exercise 2x/week + hot bathing for 20 minutes after each period of exercise; 2. 75 minutes of exercise 2x/week + dietary modification (4 nutrition guidance classes); 3. Hot bathing for 20 minutes 2x/week; 4. No TX control | ++Greater reductions in weight, BMI and body fat percentage compared to all other treatment groups and the control group | N/A | +++ |  |
| **Sanal, 2013**[55], *N*=92 overweight/obese adults; 12 week follow-up | 1. Aerobic exercise for 20–45 minutes/ day, 3-5 days/week; 2. Aerobic resistance exercise, same exercise prescription as Tx 1 + resistance training 2x/week | ++Sig reductions in weight, BMI and waist circumferences in all groups; NS differences between groups; TX 2 led to greater increases in fat-free mass than TX 1 that varied between genders: TX 1 led to greater fat free mass in the arms, trunk and whole body for men, whereas Tx 2 led to greater fat-free mass in the trunk region in women | N/A | + |  |
| **Salehpour, 2012**[121], *N*=85 overweight/ obese women; 12 week follow-up | 1. Vitamin D supplement 25 μg/day; 2. Placebo pill 25 μg/day | ++ Greater reductions in overall body mass in TX 1compared to placebo; NS differences in body weight, waist circumference or hip circumference between groups | N/A | ++ |  |
| **Sanchez, 2014**[120], *N*=125 obese adults; 12 and 24 week follow-up | 1. *Lactobacillus rhamnosus* supplement, 2x/day + moderate energy restricted diet; 2. Placebo pill, 2x/day + moderate energy restricted diet | ++TX led to greater WL at 12 and 24 week follow-ups compared with placebo | N/A | + |  |

| **Author, year, *N*, demographic, setting, follow-up** | **Intervention(s) reviewed** | **Outcomes and clinical impact** | **Adverse effects** | **Risk of bias** | |
| --- | --- | --- | --- | --- | --- |
| **Shapiro, 2012**[148], *N*=170 overweight/ obese adults; 6 and 12 month follow-up | 1. Text4Diet a daily interactive WL programme consisting of personalised text messages + monthly e-newsletter; 2. Monthly e-newsletter | + NS WL in either group at either follow-up assessment | N/A | ++ |  |
| **Shin, 2014**[117], *N*=225 obese adults; 12, 18 and 24 month follow-up | 1. Zonisamide 200mg/day + diet and lifestyle counselling; 2. Zonisamide 400mg/day + diet and lifestyle counselling; 3. Placebo pill + diet and lifestyle counselling | ++ Greater WL at 12 months in TX 2 compared with TX 1 and placebo; NS difference in WL in TX 1 and placebo; these differences were maintained at 18 month follow-up; at 24 months (6 months after the study was discontinued) patients receiving TX 2 had regained the most weight | +++ | + |  |
| **Sigal, 2014**[166], *N*=304 obese adolescents; 6 month follow-up | 1. Aerobic training 4x/week; 2. Resistance training 4x/week; 3. Combination of TX 1, 2 and 3, 4x/week; 4. No exercise | ++Sig reductions in percentage of body fat and waist circumference in all TX groups compared to no-exercise controls; NS differences between the three exercise groups | N/A | ++ |  |
| **Sijie, 2012**[57], *N*=60 overweight young women; 12 week follow-up | 1. Moderate intensity continuous training, 5x/week; 2. High intensity interval training, 5x/week; 3. No training | +++Both exercise groups led to sig reductions in body mass, BMI, body fat percentage and waist-to-hip ratio; greater reductions in body fat in TX 2 than TX 1 and no-training control | N/A | +++ |  |
| **Sovik, 2013**[138], *N*=60 morbidly obese adults; 24 month follow-up | 1. Duodenal switch; 2. Gastric bypass | +++Sig improvements in psychosocial functioning in both groups, with NS difference between groups | +++ Greater in TX 1 | ++ |  |
| **Sovik, 2011**[137], *N*=60 morbidly obese adults; 12 and 24 month follow-up | 1. Duodenal switch; 2. Gastric bypass | +++Greater reductions in BMI, total WL, waist circumference and hip circumference in TX 1 compared to TX 2; sig greater QOL improvements in TX 2, but both groups had sig improvements | +++ | ++ |  |
| **Spring, 2013**[80], *N*=69 overweight and obese adults; 3, 6, 9 and 12 month follow-up | 1. Usual physician care + mobile phone technology which participants used as a decision support tool to self-regulate energy intake; 2. Usual care control | ++Greater WL in TX 1 compared with control | N/A | ++ |  |
| **Steinberg, 2013**[146], *N*=91 overweight and obese adults; 3 and 6 month follow-up | 1. Daily self-weighing program + web-based graph of weight trends + weekly tailored feedback via e-mail + 22 weekly lessons on behavioural weight control via e-mail; 2. Delayed weighing + instruction to maintain their current self-weighing habits | +++ Greater WL at 3 and 6 months in TX 1 than control | N/A | ++ |  |
| **Strobl, 2013**[43], *N*=467 obese adults; 12 month follow-up | 1. Standard inpatient medical obesity rehabilitation + intensive after-care 2. Standard inpatient medical obesity rehabilitation | + Both groups experienced WL; the difference between groups was NS | N/A | ++ |  |
| **Suplicy, 2014**[119], *N*=174; 52 week follow-up | 1. Diethylpropion 75mg/day; 2. Fenproporex 25mg/day; 3. Mazindol 2mg/day; 4. Fluoxetine 20mg/day; 5. Sibutramine 15mg/day; 6. Placebo pill | +++TX 1, 2, 3 and 5 all led to sig WL compared to placebo, with NS differences between these drugs; all TX groups had sig improvements in QOL, depression and anxiety | ++ | +++ |  |
| **Toulabi, 2012**[165], *N*=152 obese adolescents; 6 week follow-up | 1. School-based behavioural program (nutritional, dietary and exercise education delivered to parents + exercises 3 days/week; 2. Education booklets containing information about healthy eating and exercise | ++Greater reductions in body weight, BMI and waist and hip circumference in TX 1 compared to TX 2; both groups had improvements in depressive symptoms, with NS differences between groups | N/A | +++ |  |

| **Author, year, *N*, demographic, setting, follow-up** | **Intervention(s) reviewed** | **Outcomes and clinical impact** | **Adverse effects** | **Risk of bias** | |
| --- | --- | --- | --- | --- | --- |
| **Tur, 2013**[112], *N*=106 morbidly obese adults; 1yr follow-up | 1. Intensive lifestyle intervention (including group meetings focused on dietary habits and food choices, exercise prescription and optional WL medication); 2. Conventional obesity therapy (nutritional education, medical TX and follow-up available for patients with morbid obesity) | +++ Greater WL (of more than 2, 10 and 20% of body weight) in TX 1 compared to TX 2 | N/A | ++ |  |
| **Wadden, 2011a**[111], *N*=793 overweight/ obese adults; 56 week follow-up | 1. Naltrexone SR 32 mg/day combined with bupropion SR 360 mg/day + behavioural modification; 2. Placebo pill + behavioural modification | +++ Greater WL in TX 1 compared with control: nearly twice as many individuals receiving TX 1 lost ≥10% and nearly three times as many lost ≥15% of initial weight compared with controls; TX 1 led to sig improvements in QOL compared to placebo | ++ | ++ |  |
| **Wadden 2011b**[113], *N*=390 obese adults; 24 month follow-up | 1. Brief lifestyle counselling; 2. Enhanced brief lifestyle counselling + either a meal replacement or Orlistat or sibutramine; 3. Usual primary care | ++ Greater WL at 24 months in TX 2compared with TX 1 and controls; max WL for all groups at 12 months; NS difference between addition of meal replacements, orlistat or sibutramine to counselling | ++ | ++ |  |
| **Wagener, 2012**[163], *N*=41 obese adolescents; 10 week follow-up | 1. Exergaming, a supervised 10-week group dance-base; 2. Wait list control | + NS effect of TX 1 on WL and psychological outcomes compared with control, with the exception of greater improvement in participants’ perceptions of competence | N/A | +++ |  |
| **Wang, 2015**[164], *N*=156 obese Chinese adolescents; 3 month follow-up | 1. High protein breakfast diet; 2. Standard breakfast diet | +++ Greater WL in TX 1 compared with TX 2 | N/A | +++ |  |
| **White, 2013**[114], *N*=61 overweight/ obese women with BED; 8 week follow-up | 1. Bupropion 300mg taken daily; 2. Placebo pill taken daily | ++ Greater WL after eight weeks in TX 1 compared to placebo pill controls; NS differences in psychological outcomes between groups | N/A | + |  |
| **Willis, 2012**[56], *N*=119 overweight/ obese sedentary adults | 1. Resistance training, 3x/week; 2. Aerobic training, of about 12 miles/week; 3. Aerobic training of about 12 miles/week + resistance training 3x/week | +++ Greater reductions in overall body weight, fat mass and waist circumference in TX 2 and 3 compared with TX 1; lean body mass increased sig for TX 1 and 3 | N/A | +++ |  |
| **Yeo, 2014**[143], *N*=91 overweight/ obese Korean adults; 8 week follow-up | 1. 5 point acupuncture, once/week for 8 weeks; 2. Hunger point acupuncture, once/week for 8 weeks; 3. Placebo acupuncture, once/week for 8 weeks | +++ Greater reductions in BMI at 8 weeks in TX 1 and TX 2 compared with control; NS difference between TX 1 and TX 2; sig reductions in waist circumference in all 3 groups from baseline, with greatest reduction in TX 1 | N/A | + |  |
| **Zarate, 2013**[135], *N*=43 morbidly obese; 5yr follow-up | 1. Banded RYGB; 2. Unbanded RYGB | ++ Sig WL in both groups 5 years post-surgery; NS difference between groups | ++ | ++ |  |
| **Zhang, 2014**[136], *N*=64 obese adolescents and adults; 5yr follow-up | 1. SG; 2. RYGB | ++ Max WL in both groups 1 year post-surgery, with gradual weight regain over the next 4 years; greater sustained reductions in BMI 5 years post-surgery in TX 2 compared with TX 1; sig improvements in QOL (correlated with % WL) in both groups over the 5 years post-surgery, NS difference between groups | N/A | ++ |  |

**Notes**: Only the number of RCTs is given but SRs may have included additional, non-RCT studies. The following conventions apply to all terms: Low: +, Moderate: ++, High: +++. Outcomes reflect the clinical impact of the study’s findings for physical, psychological and eating disorder (ED) outcomes. Clinical impact was ranked as low, moderate or high after assessing the statistical precision, effect size, clinical relevance and duration of impact for each study/outcome. Risk of bias was evaluated for each study using the 9-point Overview Quality Assessment Questionnaire (OQAQ). **Abbreviations**: N/A: not assessed; QOL: quality of life; Sig: statistically significant; TX: treatment; WL: weight loss. **Interventions**: BWL: behavioural weight loss; AGB: laparoscopic adjustable gastric banding; SG: laparoscopic sleeve gastrectomy; RYGB: roux-en-Y gastric bypass; VBG: vertical banded gastroplasty
