# Supplementary material for: A systematic review and narrative synthesis of interventions for uncomplicated obesity: weight loss, well-being and impact on eating disorders
Source: J Eat Disord. 2017 May 1;5:15. doi: 10.1186/s40337-017-0143-5 (PMC5410702; doi:10.1186/s40337-017-0143-5)
Supplement: Supplementary file 4 — Overview descriptions of interventions included in this review [25, 26, 179, 214–220]. (DOCX 47 kb) [file 40337_2017_143_MOESM4_ESM.docx]

**Additional file 4**

***Lifestyle interventions***

Lifestyle interventions that combined diet, exercise and behavioural changes are currently recommended as the first-line approach for the treatment of overweight and obesity[179, 214]. Lifestyle modifications involve dietary and behavioural changes that can be sustained in the long-term to promote health, and are distinct from dieting, which centres on a specific pattern of food intake over a discrete period of time.

***Dietary interventions***

Dietary interventions for overweight and obesity are designed to create a negative energy balance (e.g., energy expended is greater than energy consumed) by a sustained reduction in energy intake below an individual’s unique energy requirements[215]. Energy restricted diets include low-energy diets and very low-energy diets, that restrict kilojoules to be below the metabolic expenditure of each individual, while modified macronutrient diets alter the levels of fat, carbohydrates and proteins relative to each other The general recommendations for low-energy diets is consumption of 4200-5000 kJ for women and 5000-6700 kJ for men, with careful self-monitoring of food intake[215]. The general recommendations for very low-energy diets is consumption of 1600-3350 kJ, with large amounts of protein to preserve lean body mass.

***Exercise and physical activity interventions***

Exercise and physical activity may be prescribed alone, or in combination with behavioural and dietary interventions within a lifestyle intervention. Physical activity refers to any physical movement that utilizes one or more large muscles and increases heart rate, while exercise, a type of physical activity, refers to movement within a more structured program that maintains or enhances overall health, fitness and well-being[216]. The intensity, frequency and duration of the program are important parts of the exercise prescription.

***Behavioural and psychological Interventions***

Behavioural and psychological skill building is a cornerstone of obesity treatment. These interventions are usually delivered in group settings, and in combination with other weight loss strategies. While traditionally delivered in person, a number of recent treatment programmes have integrated technology as either the basis of treatment or as an adjunct to in-person treatment. Motivation to change and maintain positive behaviours are important factors in weight loss outcomes[217], with self-monitoring at the core of most programmes. Self-monitoring increases awareness of how behaviours are impacting weight, and creates personal accountability that fuels positive behavioural change.

***Pharmacological interventions***

Pharmacotherapies are recommended for patients who have attempted but not succeeded at attaining weight loss through lifestyle interventions[218], or delivered as an adjunct to a lifestyle intervention[25]. In Australia, the Therapeutic Goods Administration (TGA) classifies most prescription and over-the-counter medicines as registered medicines, which carry higher risk and are subject to thorough evaluation prior to reaching market[219]. The TGA classifies low-risk complimentary medicines, such as herbal medicines, vitamins, minerals and other supplements, as listed medicines, which contain only pre-approved low-risk ingredients. A variety of registered and listed medicines have been trialled for the treatment of overweight and obesity, and most of these target weight reduction by decreasing the consumption or absorption of food, and/or by increasing energy expenditure.

***Bariatric surgery***

Gastrointestinal surgery for obesity, also called bariatric surgery, alters normal digestive processes to promote weight loss and is generally reserved for individuals in the higher BMI range (>40 kg/m2). Bariatric surgery procedures include two classes of surgery: (1) malabsorptive procedures, which limit the absorption of calories, proteins and nutrients, and (2) restrictive procedures, which restrict gastric volume, and consequently, the amount of food that can be consumed[26]. In Australia, the three most commonly performed bariatric procedures are laparoscopically performed Roux-en-Y gastric bypass (RYGB), sleeve gastrectomy and adjustable gastric banding[25]. Bariatric surgery reduces weight by reducing hunger, increasing satiety, restricting food intake or causing a malabsorption of food[26] via surgical alterations to the gastrointestinal tract[26, 220].
